# An Interprofessional Faculty Development Program for Workplace-Based Learning

**DOI:** 10.5334/pme.1242

**Published:** 2024-05-01

**Authors:** Eveline Booij, Marjel van Dam, Gersten Jonker, Lisette van Bruggen, Marije Lesterhuis, Marieke F. van der Schaaf, Reinier G. Hoff, Marije P. Hennus

**Affiliations:** 1 Department of Anesthesiology, University Medical Center Utrecht, Utrecht, the Netherlands; 2 Intensive Care Center, University Medical Center Utrecht, Utrecht, the Netherlands; 3 Utrecht Center for Research and Development of Health Professions Education, University Medical Center Utrecht, Utrecht, the Netherlands; 4 Pediatric Intensive Care, Wilhelmina Children’s Hospital, University Medical Center Utrecht, Utrecht, the Netherlands

## Abstract

**Background:**

Most faculty development programs in health professions education, pivotal in cultivating competent and effective teachers, focus on systematic, planned and formal learning opportunities. A large part of clinical teaching however, encompasses ad-hoc, informal and interprofessional workplace-based learning whereby individuals learn as part of everyday work activities. To fully harness the educational potential embedded in daily healthcare practices, prioritizing interprofessional faculty development for workplace-based learning is crucial.

**Approach:**

Utilizing the ‘ADDIE’ instructional design framework we developed, implemented and evaluated an interprofessional faculty development program for workplace-based learning. This program, encompassing seven formal training sessions each with a different theme and five individual workplace-based assignments, aimed to support clinical teachers in recognizing and optimizing informal learning.

**Outcomes:**

The pilot program (n = 10) and first two regular courses (n = 13 each) were evaluated using questionnaires containing Likert scale items and open textboxes for narrative comments. The quality and relevance of the program to the clinical work-place were highly appreciated. Additional valued elements included practical knowledge provided and tools for informal workplace-based teaching, the interprofessional aspect of the program and the workplace-based assignments. Since its development, the program has undergone minor revisions twice and has now become a successful interprofessional workplace-based alternative to existing faculty development programs.

**Reflection:**

This faculty development program addresses the specific needs of healthcare professionals teaching in clinical settings. It stands out by prioritizing informal learning, fostering collaboration, and supporting integration of formal training into daily practice, ensuring practical application of learned knowledge and skills. Furthermore, it emphasizes interprofessional teaching and learning, enhancing workplace environments.

## Background & Need for Innovation

Faculty development, pivotal in shaping competent and effective teachers, refers to a wide range of activities employed by institutions to support staff in their teaching roles [1,2]. Notably, most faculty development programs in health professions education primarily consist of systematic formal learning opportunities that involve planning, implementation and evaluation of teaching [3]. One form of faculty development that has been adopted by all Dutch universities following its introduction by Utrecht University in 1996, is the University Teaching Qualification (UTQ). Since 2008, this quality mark of academic teaching is obligatory for all university teachers. The UTQ aims to improve faculty members’ teaching competencies based on learning objectives matching the framework for University Teachers [4]. A combination of courses, assignments, observations in their educational practice and reciprocal exchange is used to train teachers in the UTQ program in: i) educational design, including the formulation of learning goals and the development of learning tasks, instructional methods, and assessment; ii) executing education by giving lectures and seminars, and by mentoring individuals and groups of students; and iii) the evaluation and furthering of their own professional development as an educator.

In a commendable initiative to support faculty development in the clinical setting, several University Teaching Hospitals, including the University Medical Center Utrecht (UMCU), have obligated their physicians to obtain an UTQ when teaching at least half a day per week. Over the years though, formal and curbside feedback pointed towards a misalignment between the UTQ program and the needs, demands and daily teaching practice of healthcare professionals. The main focus of the UTQ, is on formal teaching in a classroom setting (e.g., giving lectures, designing a course and exam questions). A large part of the UMCU faculty’s teaching however, encompasses ad-hoc and/or informal and mostly interprofessional learning situations in which individuals or teams teach and learn as part of their everyday work activities [5,6]. This mismatch was found to be worrisome, as alignment to the clinical setting is a key factor in fostering transfer of knowledge and skills from faculty development to sustainable educational practice [1,7,8,9]. Furthermore, although the benefits of informal learning have been described and embraced by health profession educators [2,10,11], students and teachers do not always recognise informal learning as education thereby potentially attenuating its impact [12].Subsequently, to fully harness the educational potential inherent in daily healthcare practices, it is essential to prioritize interprofessional faculty development for informal workplace-based learning [13].

## Goal of Innovation

To better align faculty development with the clinical teaching setting of healthcare professionals and optimize informal workplace-based teaching, a new faculty development program was created. This program, aptly titled the ‘Clinical Teaching Qualification’ (CTQ), was collaboratively developed by medical educators, clinicians, and educationalists at the UMCU. The goal of the CTQ is to teach clinical teachers how to recognize and optimize formal (supervision, feedback, development conversations, etc.) and informal (while performing daily care related tasks) teaching situations. Furthermore, to reflect and underpin the interprofessional aspect of teaching and learning in most healthcare settings, as well as to acknowledge and foster the educational qualities of non-physicians, the CTQ is intended for all healthcare professionals (physicians, nurses, physician assistants, etc.) with workplace-based teaching as a significant part of their professional task. Finally, to stimulate transfer of learning from the formal training program to daily practice, so-called transfer tasks were used.

## Steps Taken for Development and Implementation of Innovation

This paper is a descriptive account of the process through which the CTQ program was developed and implemented utilizing the ADDIE (Analysis, Design, Development, Implementation and Evaluation) instructional design framework [14]. In short, learning outcomes of the CTQ were determined, and attractive learning activities were designed. Appropriate assessment methods were selected and purposefully connected to learning activities and intended learning outcomes to ensure constructive alignment. Finally, evaluation of the pilot program and first two regular courses resulted in two consecutive minor revisions of the CTQ program, leading to its current iteration.

### Analysis

First, we conducted a needs assessment in which we interviewed twelve stakeholders from various healthcare disciplines. This group was purposefully sampled and consisted of experienced workplace supervisors, clinical teachers and post-graduate course directors from (para)medical and nursing programs. To complete the needs assessment, two key educational managers and researchers were consulted to solicit their insights and feedback on the interview reports, thereby contributing to a comprehensive understanding of the new faculty development program. Throughout the design process, active engagement of all stakeholders was sustained, as they were consistently invited to provide feedback on (various parts of) the concept design.

### Design

Based on the results from the needs assessments the UTQ learning objectives were thoroughly revised by a team of educationalists and medical educators to better fit informal workplace-based teaching, which resulted in specific learning objectives for the new CTQ (supplemental Table 1).

**Figure 1:**
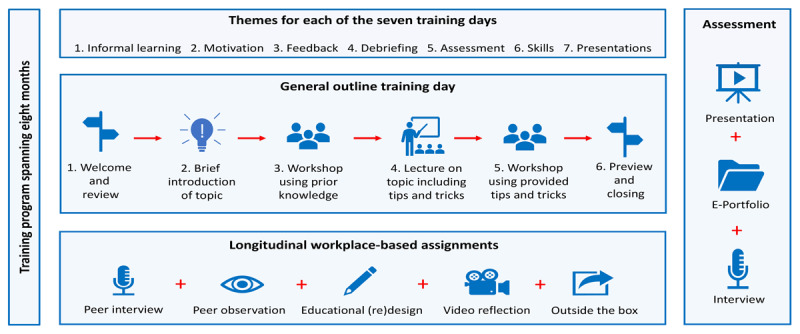
**Visual representation of the content of the Clinical Training Program**. The Clinical Training program teaches teachers how to recognize and support informal learning of students during daily clinical practice using a balanced mix of lectures, workshops and interprofessional workplace-based assignments revolving around seven themes.

The core of the new faculty development program revolves around seven formal training sessions each with a different theme: i) Recognize and optimize informal interprofessional learning opportunities; ii) Motivate learners with help of the self-determination theory and explore the hidden curriculum of a workplace; iii) Operationalize constructive interprofessional feedback dialogues; iv) Use and optimize team debriefings as learning opportunities; v) Use and critically analyze various forms of workplace-based assessment; vi) Teach technical and non-technical skills and; vii) Presentations of participants’ main learning points during the program (Figure 1).

### Development

The first six training sessions have the same educational design: a local healthcare professional with expertise in the theme of the relevant training session is invited to give a guest lecture and teams up with the clinician educator and educationalist leading the program. Using a flipped classroom approach, participants prepare for each training session by watching a video and/or reading provided articles. At the start of the training session, the guest lecturer briefly introduces the theme, after which participants perform a relevant small group activity using their prior knowledge and skills on the topic. This inductive learning method activates prior knowledge and allows participants to explore and pinpoint specific and/or personal learning needs [15]. After a brief group reflection on the activity, the guest lecturer then elaborates on the theme using an interactive lecture and provides participants with practical tips and tricks. Next, participants repeat the earlier activity, this time applying their newly acquired knowledge and skills. At the end of each training session, participants reflect on what element appealed most to them and make explicit how they plan to implement that specific element in their own daily practice, preferably the next day at work, by filling out the so-called transfer tasks. Making one’s intention to apply the learned knowledge and/or skills explicit, promotes transfer of learning from the training setting to daily practice [16]. The following training session, approximately one month later, starts with participants filling out the second half of the transfer task, reflecting individually on their intended goals from the previous session. Thereafter a group discussion on potential facilitators and barriers in reaching those goals is held before a new theme is started. This group reflection not only strengthens particpants own learning but also allows for sharing of and learning from other experiences [17]. On the final training day, participants share and reflect on their main learning points as well as their future goals during a ten-minute oral presentation on which they receive feedback on both form and content from peer participants and faculty. Participants’ presentations alternate with two interactive lectures on a topic chosen by the participants earlier during the program. To further support participants’ learning and to help build a community of practice, three of the seven training sessions include group intervision sessions, a form of peer learning that helps create a safe and reflective space amongst teams to tackle common challenges or problems [18].

These seven formal training sessions are spread over an eight-month period to allow time for sinking-in and reflection and are supplemented with five individual workplace-based assignments that align with the content of the CTQ. These assignments include: 1) Interviews with three interprofessional colleagues in the participant’s work environment about their perspective on informal learning (what is it, how is it used, who is involved etc.); 2) Observing a teaching activity of a peer CTQ participant and providing feedback; 3) Designing or revising an educational activity using the educational design principles; 4) Video recording one’s teaching activity and sharing that recording with the other participants during training day six in order to receive feedback on one’s teaching; and 5) Taking the opportunity to explore the world of healthcare education beyond the participant’s own frame of reference for example by attending an educational conference or a clinical competency committee meeting of another training program etc.

After completion of the program, participants are assessed based on their e-portfolio (including a recording of their presentation, their educational resume, reflections on learning, completed workplace-based assignments and received interprofessional feedback) by faciliators of the UMCU Center for Research and Development of Health Professions Education and a formal interview with two members of the board of assessors of the UTQ. If CTQ objectives are noticeably met and all elements of assessment are deemed sufficient, the official CTQ-certificate is granted to the participant.

### Implementation

A pilot version of the CTQ training program was conducted in 2021 with ten purposefully selected participants (four nurses, one physician assistant and five physicians), all experienced healthcare professionals and educators with workplace-based teaching as an essential part of their daily teaching practice. Thereafter, two ‘regular’ courses (in 2022 and 2023) were held with a balanced mix of physicians and allied health care professionals (n = 13 per course).

## Outcomes of Innovation

### Evaluation

**Figure 2:**
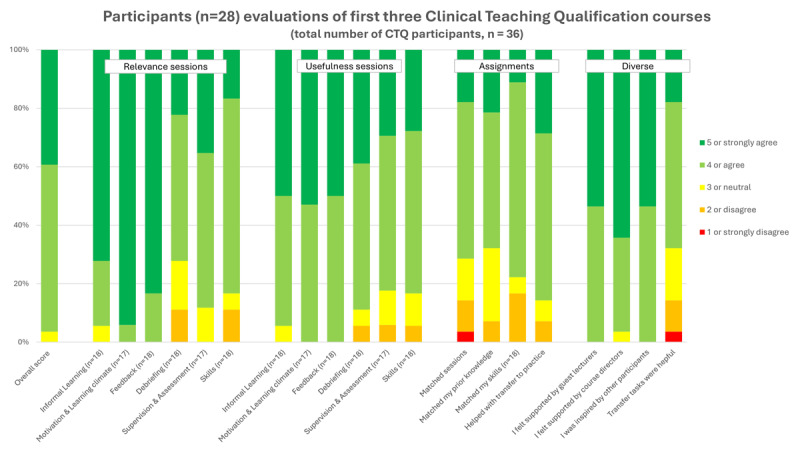
**Evaluation scores of the pilot and first 2 regular Clinical Teaching Qualification courses**. Five-point Likert-scale data for various aspects of the program, including the program as a whole, the relevance and usefulness of each session, assignments, the support experienced from lecturers and course directors, inspiration derived from peer participants, and the helpfulness of transfer tasks. The number of respondents for each item was 28 unless stated otherwise.

As the final step of the ADDIE design model, we evaluated the pilot CTQ program (n = 10) and the first two ‘regular’ courses (n = 26) using questionnaires. These questionnaires consisted of five-point Likert scale items assessing various aspects including the overall program, relevance and usefulness of each session, alignment between assignments and sessions, facilitation of transfer to daily practice, perceived support from lecturers and course directors, inspiration from interprofessional peers, effectiveness of transfer tasks, as well as open text boxes for narrative comments on all elements of the program. Calculating the average and Cronbach’s alpha for a scale was not possible given the too few individuals in certain groups, violating the assumption of a sufficiently large sample size per group, which could lead to unreliable estimates and compromised statistical validity. Subsequently, medians were calculated for the Likert scale data, with a predetermined consensus threshold of 4 (out of the 5-point Likert scale, where levels 4 and 5 correspond to the two highest agreement levels) indicating a need for revision for the respective item. The results show that all items attained a median score of 4 or above (supplemental Table 2). Moreover, the quality (overall score), and relevance and usefulness (although the latter two were not individually assessed for each session in the pilot) of the CTQ program in the clinical workplace were widely acknowledged, as indicated by predominantly high Likert scores of 4 or higher (Figure 2). Additional valued elements were the practical knowledge provided and tools for informal workplace-based teaching, the interprofessional aspect of the program, the intervision and the workplace-based assignments (Figure 2). This was further supported by narrative comments, e.g.: “*There was a good connection between education and work in the clinic – truly a niche that was missing. Lots of practical and very fitting tools to get started with”; “I really valued the interdisciplinary aspect and feel that it has great added value. Everyone has a different perspective after all” and; “Integration into my daily clinical practice was supported by both the intervision and the transfer tasks”*.

Suggestions for improvement from the narrative feedback included better alignment of the workplace-based assignments with the relevant sessions and more time to further explore featured themes, e.g., *“Sometimes, I found that the meetings were a bit too short, not so much in terms of time but in terms of content”*

The narrative feedback further indicated a wide variation in what participants took from the program and actually implemented in their daily clinical teaching practice. This ranged from increased awareness about featured themes for example: “*I have sharpened my teaching techniques. I now use ‘debriefing with good judgment’ and have learned how to integrate Socratic questioning. I am also much more aware of the ‘hidden curriculum’ and my continuous role as an educator”;* to implementation of content like recognizing and optimizing informal learning moments during daily work and ensuring change in a medical department by the introduction of an e-portfolio for assessment of junior house officers. The latter was the result of the ‘Re-design workplace-based assignment’ of one of the participants. Perceived barriers to implementation of training content were a lack of time, not having enough exposure to learners to practice CTQ-training content, and a lack of autonomy expressed as the feeling of inability to change day-to-day business. Notably, this evaluation focused on the lower levels of Kirkpatrick’s model for evaluating the efficacy of a training program, namely participants’ reaction to and perceived learning from the program [19].

Based on the evaluation results of the pilot and the initial two regular courses, the CTQ training program underwent minor revisions twice, based on the narrative feedback. An additional session was incorporated to afford time for comprehensive coverage of the existing content, while workplace-based assignments were meticulously realigned with their respective sessions, with clear introductions and explanations provided during these sessions. This program has since evolved into the workplace-based counterpart of the UTQ, becoming an integral and highly sought-after component of the UMCU Faculty Development program, offered once or twice annually.

## Critical Reflection on the Process

The newly developed interprofessional CTQ aligns with the needs and demands of healthcare professionals tasked with teaching learners in the complex setting of a clinical environment. It distinguishes itself from other faculty development programs through its emphasis on prioritizing informal learning and teaching, acknowledging that the majority of health professionals’ learning transpires in informal settings. Moreover, it encourages collaborative and interprofessional learning through its structure, the use of intervision and facilitates rolemodelling interprofessional teaching and learning into workplace environments. Additionally, it stimulates the seamless integration of formal training into daily practice through transfer tasks, ensuring practical application of acquired knowledge and skills. Subsequently, our program could serve as an example for other institutions aiming to optimize faculty development for clinical teachers in the provision of workplace-based teaching and assessment.

The process of development of this program has yielded both strengths and limitations. One of the key strengths of the program is its ability to provide faculty with practical tools to recognize and optimize their informal teaching in the time-pressed interprofessional clinical setting. The healthcare environment is often fast-paced, leaving little room for formal teaching activities. By emphasizing the importance of informal teaching and equipping faculty with strategies to incorporate this into daily practice without taking much time out of their clinical responsibilities, the program addresses a crucial need. This can lead to more effective and efficient knowledge transfer among healthcare professionals, ultimately benefiting patient care. Another strength lies in the development of an interprofessional teacher community. The formation of a community can enhance interprofessional teamwork and improve communication among healthcare professionals. Learners react positively to interprofessional education (IPE), with reported improvements in attitudes, perceptions, collaborative knowledge, and skills [20]. Nonetheless, there is still limited evidence regarding the effects of IPE on behavior change, organizational practice, and patient outcomes. Finally, the CTQ program demonstrates the potential to increase intrinsic motivation among participants to use informal teaching in the workplace. By designing tasks and activities that capture participants’ enthusiasm and align with their professional goals, the program promotes a sense of ownership and empowerment [21]. This heightened intrinsic motivation can translate into a sustained commitment to informal teaching practices, benefiting both the learners and the broader healthcare team [22]. Whether this is indeed the case, is the topic of ongoing studies.

The program is not without limitations. The small scale of the program restricts its reach and impact. While the focused approach allows for personalized support and attention, it may limit the number of participants who can benefit from the program. Scaling up the program to accommodate a larger audience will require careful planning and resource allocation. In addition, the tuition fee associated with the program poses a potential barrier to participation. Financial constraints can prevent healthcare professionals from accessing professional development opportunities [23]. It is important to consider strategies to mitigate these barriers, such as seeking funding or exploring alternative models for program delivery that minimize financial burdens. Interestingly, the success of the program has led to an unexpected challenge: demand now exceeds availability. This highlights the program’s perceived value and recognition of its effectiveness within the healthcare community. Nevertheless, it also necessitates careful management to ensure equitable access for all interested participants. A related limitation pertains to the potential inclusion of residents, student nurses or trainee physician assistants who are often tasked with supervising junior learners like interns. Even so, trainees often have limited experience with workplace-based teaching and lack formal training in teaching methods. A certain level of maturity in one’s own field and in workplace-based teaching is necessary to fully benefit from the interprofessional and practice-oriented approach of the CTQ. As such, enrolling trainees into the current CTQ program is challenging. Finally, achieving success in this program requires a significant investment of time, both in terms of direct engagement and completing assignments within the workplace. This demand for time can pose a challenge for healthcare professionals who are already facing time constraints.

In conclusion, the Clinical Teaching Qualification (CTQ) program offers a tailored solution to address the gap between formal faculty development and the demands of clinical teaching. By prioritizing informal learning and interprofessional collaboration, it equips healthcare professionals with practical tools to enhance teaching in the clinical setting. While the program demonstrates promising outcomes, including improved awareness of informal teaching and strengthened interprofessional networks, challenges such as scalability and accessibility remain. Further research and adaptation are needed to ensure broader participation and sustained impact in clinical education. Ensuring the consolidation and long-term sustainability of the program are critical factors for successful implementation and ongoing growth within the learning community. By leveraging strengths and proactively addressing limitations, the program is well-positioned to make a significant contribution to advancing interprofessional workplace-based teaching.

## Additional File

The additional file for this article can be found as follows:

10.5334/pme.1242.s1 Supplemental data. Supplemental Tables 1 and 2.
