# Supplementary material for: An Interprofessional Faculty Development Program for Workplace-Based Learning
Source: Perspect Med Educ. 2024 May 1;13(1):266–73. doi: 10.5334/pme.1242 (PMC11067978; doi:10.5334/pme.1242)
Supplement: Supplemental data. — Supplemental Tables 1 and 2. [file pme-13-1-1242-s1.pdf]

Supplemental data

Supplemental table 1: Learning outcomes of the Clinical Teaching Qualification

**I – Professional development**	
Area of competence	The clinical teacher is able to:
I a – Expertise	• keep professional knowledge and skills up to date;<br>• master the current subject content in such a way that he/she can apply it in the design of learning in the workplace.
I b – Didactic knowledge	• keep didactic knowledge and skills to promote workplace-based learning up-to-date;<br>• implement local training plans as intended.
I c – Developmental	• reflect on one's own functioning and development as a clinical teacher;<br>• seek additional feedback and convert this input into new actions/experiments;<br>• give colleagues education- and training-related (peer) feedback;<br>• learn from interprofessional feedback and from patient feedback.
I d – Role model	• promote the importance of training;<br>• play an active role in the continuous development of the training;<br>• connect and collaborate interprofessionally in education;<br>• use patient participation in education<br>• promote inclusivity and diversity;<br>• contribute to a safe and stimulating working environment for learners.

**II – Performing training tasks in the clinic**	
Area of competence	The clinical teacher is able to:
II a – Training in the workplace	• translate learning theories of adult learning into a clinical context;<br>• apply didactic principles of adult learning in a clinical context;<br>• use a variety of teaching methods and learning materials, appropriate to learning objectives;<br>• fulfil different roles, appropriate to the chosen learning activity;<br>• recognize and exploit learning opportunities in the workplace, appropriate to the level of competence and independence of the student/assistant;<br>• create a stimulating, activating, safe and motivating learning environment.
II b – Supervising students and residents	• apply the basic principles of coaching in guiding an individual learning journey of students/assistants in the workplace.<br>• supervise assignments of students/residents
II c – Team training	• work (interprofessionally) in a team and consult with colleagues and/or other relevant persons about the content and form of workplace-based learning and training of students/residents

**III – Feedback and assessment**	
Area of competence	The clinical teacher is able to:
III a – Giving and assessing feedback	• make asking for feedback and assessment accessible to students/residents by creating a safe learning environment<br>• apply the principles of constructive feedback to the clinical workplace;<br>• when giving feedback and assessments align them with the content of the programme and individual learning objectives of the student/resident;<br>• apply the preconditions of fair assessment when providing feedback and assessments (valid, free of bias, appropriate to shared frames of reference, etc.);<br>• use assessment tools and guidance tools correctly;<br>• (together with the training group) to assess various (part task)

	performances and to translate them into a final assessment, such as a positive or negative opinion regarding a competency decision.

**IV – Designing workplace-based training**	
Area of competence	The clinical teacher is able to:
IV a – Designing learning situations	• identify, further analyse, elaborate and shape opportunities for (re)design to improve the programme, taking into account the differences between students/residents
IV b – Evaluative design	• evaluate your own (re)design, interpret educational evaluations and convert them into improvement proposals;<br>• converse with committees about the quality assurance cycle, for example the programme committee;<br>• explore the possibilities for further implementation of the innovation;<br>• use the PDCA cycle when designing and implementing innovative medical education in the workplace.
IV d – Renew	• integrate current developments in the field and medical education into their own (re)design.

**V – Contribution to knowledge and impact**	
Area of competence	The clinical teacher is able to:
V a – Outreach	• share findings through outreach activities (e.g. in the training region or in the profession) and thus contribute to knowledge about clinical education.
V b – Inreach	• actively participate in peer exchange of educational experiences, ideas and evidence of successful improvements in education.

Supplemental table 2: Likert scale data of program evaluation

			Relevance							Usefulness													
**Year**	**N participants**	**Nr Evaluations**	**Overall**	**Informal learning**	**Motivation & Learning climate**	**Feedback**	**Debriefing**	**Supervision & Assessment**	**Skills**	**Informal learning**	**Motivation & Learning climate**	**Feedback**	**Debriefing**	**Supervision & Assessment**	**Skills**	**Assignments matched sessions**	**Assignments matched prior knowledge**	**Assignments matched participants' skills**	**Assigments helped with transfer**	**Support by lecturers**	**Support teachers**	**Inspired by other participants**	**Helpfulness TT**
2021	10	10	5													5	5		5	5	5	5	5
			5													5	5		4	5	5	5	4
			4													4	4		4	5	5	5	4
			5													5	5		5	5	5	5	5
			4													4	4		4	4	4	5	2
			4													4	2		4	4	4	4	4
			5													4	5		5	4	4	4	3
			5													5	5		5	5	5	5	3
			5													4	4		4	5	5	5	5
			5													5	4		5	4	5	5	1
2022	13	8	4	5	-	5	4	4	4	5	-	5	4	4	4	4	4	4	4	4	4	4	4
			4	5	5	5	3	4	4	4	4	4	3	3	5	3	3	3	4	5	5	5	5
			4	4	4	4	3	4	4	4	4	4	4	4	4	2	2	2	4	4	4	4	4
			3	5	5	5	5	-	4	5	5	5	5	-	4	1	3	2	3	4	5	4	2
			5	3	5	5	2	3	2	3	4	4	2	2	2	4	4	4	4	5	5	5	4
			4	5	5	4	4	5	3	4	4	4	5	4	4	3	4	4	4	4	4	4	4
			4	5	5	5	5	5	5	5	5	5	5	5	5	4	5	5	4	5	5	5	4
			4	5	5	4	3	4	4	5	4	4	4	4	4	2	3	4	5	4	3	5	2
2023	13	10	5	5	5	5	4	5	4	5	5	5	5	5	4	4	3	4	4	5	5	4	4
			4	5	5	5	4	4	4	5	5	5	4	4	3	3	3	4	5	5	4	5	3
			4	4	5	5	5	4	4	4	5	4	4	4	4	4	4	4	4	4	4	4	3
			5	5	5	5	4	3	4	5	5	5	4	3	4	4	3	4	4	5	5	4	3
			4	4	5	5	4	4	2	4	4	4	4	4	3	3	4	4	2	4	4	4	4
			5	5	5	5	4	5	4	5	5	5	5	5	5	4	3	4	4	5	5	5	5
			4	5	5	5	2	5	5	4	4	4	4	4	4	2	4	2	2	4	5	4	4
			4	5	5	5	5	4	5	4	5	5	5	5	5	4	4	5	3	5	5	5	4
			4	5	5	5	4	5	4	5	5	5	5	5	5	4	4	4	5	4	5	4	4
			4	4	5	5	4	4	4	4	4	4	4	4	4	4	4	4	4	5	5	4	4
**Median**			**4**	**5**	**5**	**5**	**4**	**4**	**4**	**4,5**	**5**	**4,5**	**4**	**4**	**4**	**4**	**4**	**4**	**4**	**5**	**5**	**5**	**4**
**Min**			**3**	**3**	**4**	**4**	**2**	**3**	**2**	**3**	**4**	**4**	**2**	**2**	**2**	**1**	**2**	**2**	**2**	**4**	**3**	**4**	**1**
**Max**			**5**	**5**	**5**	**5**	**5**	**5**	**5**	**5**	**5**	**5**	**5**	**5**	**5**	**5**	**5**	**5**	**5**	**5**	**5**	**5**	**5**
Total	36	28	4,4	4,7	4,9	4,8	3,8	4,2	3,9	4,4	4,5	4,5	4,2	4,1	4,1	3,7	3,8	3,7	4,1	4,5	4,6	4,5	3,7

Five-point Likert-scale data for the program as a whole (1= strongly disagree, 2 = disagree, 3 = neutral, 4 = agree, 5 = strongly agree); relevance and usefulness of each of the sessions; assignments; experienced support by lecturers and course directors; inspiration by peer participants; and helpfulness of transfer tasks. Sessions were not individually evaluated for the pilot.
